# Assessment of myocardium at risk in non-ST-elevation infarction by cardiac magnetic resonance imaging and invasive angiographic validation by the APPROACH-score

**DOI:** 10.1186/1532-429X-16-S1-P194

**Published:** 2014-01-16

**Authors:** Dominik Buckert, Vinzenz Hombach, Volker Rasche, Jochen Wöhrle, Wolfgang Rottbauer, Peter Bernhardt

**Affiliations:** 1 University of Ulm, Ulm, Germany

## Background

In the setting of acute myocardial ischemia, there is a hypoperfused portion of the myocardium that is in danger of becoming irreversibly injured. This portion of myocardium is often referred to as area at risk (AAR). The aim of our trial was to perform T2-weighted cardiac magnetic resonance imaging (CMR) for assessment of AAR in patients presenting with acute non-ST-elevation myocardial infarction (NSTEMI) and to validate this approach against the established APPROACH-score as assessed by coronary x-ray angiography.

## Methods

We enrolled sixty-four patients presenting with acute NSTEMI that underwent coronary x-ray angiography within 72 hours of symptom onset. Two blinded readers performed offline angiographic AAR assessment using the modified APPROACH-score, as being described elsewhere. For measurement of AAR by CMR, a 1.5 T whole-body scanner with a 32-channel phased-array surface coil was used. Besides functional and volumetric analyses assessed by standard steady-state free-precession sequences, a 3D T2-weighted black-blood fat-saturated spin-echo sequence was used for visualization of myocardial edema. A cut-off signal intensity increase of more than two standard deviations of remote myocardium was used for the definition of edema. Area at risk was calculated as the resulting edema volume in relation to left ventricular mass. Using this approach, AAR was quantified by two fully blinded readers.

## Results

The resulting mean AAR determined by the modified APPROACH-score was 28.6 ± 10.0%. The mean CMR derived AAR was 27.9 ± 13.7%. CMR assessment tended to slightly underestimate the AAR in comparison to angiographic scoring (difference -0.21 ± 8.1%). There is a good correlation between the AAR assessed by CMR and by angiography (r = 0.84, p < 0.0001).

## Conclusions

T2-weigthed CMR is able to quantify the AAR with very good correlation to the angiographic APPROACH-score in NSTEMI patients. Therefore, CMR might serve as excellent surrogate in clinical reperfusion trials.

## Funding

None.

